# Striatal CDK5 Regulates Cholinergic Neuron Activation and Dyskinesia-like Behaviors through BK Channels

**DOI:** 10.34133/research.0121

**Published:** 2023-04-18

**Authors:** Chu Tong, Peng-Xiang Min, Qian Zhang, Ru-Xin Gu, Yao-Hai Wen, Yi Shi, Yu-Huan Bao, Xiang Chen, Yi-Xuan Zhang, Xing-Feng Mao, Hao-Yang Yuan, Xiu-Xiu Liu, Takuya Sasaki, Li Zhang, Feng Han, Ying-Mei Lu

**Affiliations:** ^1^ Department of Physiology, School of Basic Medical Sciences, Nanjing Medical University, Nanjing, 211166, China.; ^2^ International Joint Laboratory for Drug Target of Critical Illnesses, School of Pharmacy, Nanjing Medical University, Nanjing, 211166, China.; ^3^ Department of Geriatrics, Nanjing Brain Hospital Affiliated to Nanjing Medical University, Nanjing, 210029, China.; ^4^ Institute of Pharmacology and Toxicology, College of Pharmaceutical Sciences, Zhejiang University, Hangzhou, 310058, China.; ^5^ Gusu School, Nanjing Medical University, Suzhou Municipal Hospital, The Affiliated Suzhou Hospital of Nanjing Medical University, Suzhou, 215002, China.; ^6^ Department of Pharmacology, Graduate School of Pharmaceutical Sciences, Tohoku University, Sendai, 980-8578, Japan.; ^7^ Institute of Brain Science, the Affiliated Brain Hospital of Nanjing Medical University, Nanjing, 211166, China.

## Abstract

Disturbance of the cholinergic system plays a crucial role in the pathological progression of neurological diseases that cause dyskinesia-like behaviors. However, the molecular mechanisms underlying this disturbance remain elusive. Here, we showed that cyclin-dependent kinase 5 (*Cdk5*) was reduced in cholinergic neurons of midbrain according to the single-nucleus RNA sequencing analysis. Serum levels of CDK5 also decreased in patients with Parkinson’s disease accompanied by motor symptoms. Moreover, *Cdk5* deficiency in cholinergic neurons triggered paw tremors, abnormal motor coordination, and motor balance deficits in mice. These symptoms occurred along with cholinergic neuron hyperexcitability and increases in the current density of large-conductance Ca^2+^-activated K^+^ channels (BK channels). Pharmacological inhibition of BK channels restrained the excessive intrinsic excitability of striatal cholinergic neurons in *Cdk5*-deficient mice. Furthermore, CDK5 interacted with BK channels and negatively regulated BK channel activity via phosphorylation of threonine-908. Restoration of CDK5 expression in striatal cholinergic neurons reduced dyskinesia-like behaviors in *ChAT-Cre*;*Cdk5^f/f^* mice. Together, these findings indicate that CDK5-induced phosphorylation of BK channels involves in cholinergic-neuron-mediated motor function, providing a potential new therapeutic target for treating dyskinesia-like behaviors arising from neurological diseases.

## Introduction

Dyskinesia-like behaviors are common neurological disorders occurring in neurological diseases such as Parkinson’s disease (PD), ataxia, dystonia, and Huntington’s disease [1,2]. Abnormal corticothalamic projections to the striatum contribute to the pathophysiology of these movement syndromes [3,4]. The striatum is required for motor planning, sequencing, and execution [5]. Within the striatal circuit, which is associated with the control of motor coordination and cognitive function, cholinergic neurons play an important regulatory role [6,7].

Increased cholinergic activity and acetylcholine levels are characteristics of PD and influence the functional output of striatal circuits [8,9]. However, anticholinergics, including trihexyphenidyl and procyclidine, are not currently commonly used to treat dyskinesia-like behaviors in PD due to their adverse effects, such as confusion and hallucinations [10,11]. As these traditional anticholinergic drugs indirectly inhibit central hypercholinergic excitability by extensively blocking central acetylcholine receptor or reducing acetylcholine [11], a new motor-function-related target is needed to precisely regulate cholinergic activity.

Striatal cholinergic neurons have extensive axonal fields that project locally to affect the output of striatal projection neurons [12], which display specific firing properties of tonic firing with a transient pause [13]. Striatal cholinergic neurons also show 2 periodic patterns: rhythmic bursts and single spikes [14,15]. Aberrant K^+^ channels activity alters the excitability of striatal cholinergic neurons, which is associated with pathological process of dyskinesia and PD. For example, the decreased Kv1.3 currents induce hyperexcitability of cholinergic neurons in the striatum of PD mice [16]. Although previous findings have suggested that delayed rectifier, hyperpolarization-activated, and calcium-dependent K^+^ channels contribute to the spontaneous firing patterns of striatal cholinergic neurons [14,15,17], studies have yet to identify the precise biochemical substrate that modifies electrophysiological currents or explains these changes.

Cyclin-dependent kinase 5 (CDK5) is a serine/threonine protein kinase involved in a variety of critical functions related to neural signaling pathways and neuronal activity [18,19]. For example, CDK5 suppresses both presynaptic dopamine release and postsynaptic dopamine signaling in striatal neurons [20,21]. In addition, specific knockdown of *Cdk5* in the dorsal striatum disrupted the excitatory/inhibitory synaptic balance [19]. Furthermore, medium spiny neurons of the nucleus accumbens in *Cdk5*-conditional knockout (cKO) mice exhibited elevated intrinsic neuronal activity [22]. Therefore, it is essential to establish the causality in the association between CDK5 signaling and cholinergic neuronal activity; hence, an approach will provide a viable rescue strategy to reverse the cellular adaptations and behavioral symptoms of neurological disorders.

Here, we report the potential roles of CDK5 in regulating striatal cholinergic neuron activity via Ca^2+^-activated K^+^ channels (BK channels). Loss of cholinergic CDK5 induces hyperactivity of striatal cholinergic neurons and abnormal motor function in mice. Combining gene technology with electrophysiology, we demonstrate that CDK5 binds to BK channels and limits BK-mediated currents by phosphorylating threonine-908 (T908). More importantly, restoration of CDK5 expression in striatal cholinergic neurons alleviated the motor dysfunction induced by cholinergic-neuron-specific *Cdk5* deficiency. Together, our pharmacological and genetic findings suggest that CDK5 signaling may represent a viable target to ameliorate dyskinesia-like disorders.

## Results

### Decreased CDK5 mRNA and serum CDK5 in PD patients

**Fig. 1.**
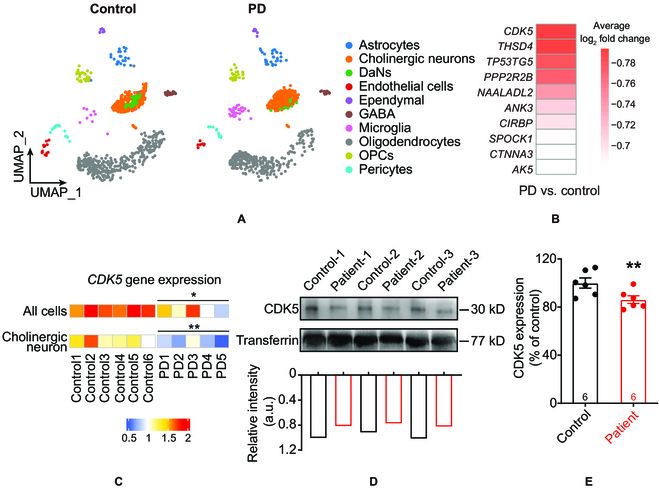
Decreased *CDK5* mRNA and serum CDK5 levels in patients with PD. (A) Uniform manifold approximation and projection (UMAP) plot of all 41,427 midbrain cells from HCs and patients with PD. Colors indicate different cell types. (B) The top 10 downregulated genes in patients with PD according to model-based analysis of single-cell transcriptomics. (C) The mRNA expression of *CDK5* in patients with PD decreased in all midbrain cell types and especially in cholinergic neurons compared with that of HCs. ^*^P < 0.05 and ^**^P < 0.01, unpaired 2-tailed Student’s *t* test. Data represent the means ± SEM. (D and E) Representative bands of WBs and quantification of CDK5 in the serum of patients with PD (*n* = 6) and HCs (*n* = 6), ^**^P < 0.01, unpaired 2-tailed Student’s *t* test. Data represent the means ± SEM. DaNs, dopaminergic neurons; GABA, GABAergic neurons; OPCs, oligodendrocyte precursor cells; a.u., arbitrary units.

Lesions of cholinergic systems contribute to motor and nonmotor syndromes in PD [9]. Here, we filtered 41,435 cells from a Gene Expression Omnibus dataset (GSE157783) through quality control; this dataset contains single-nucleus RNA sequencing (snRNA-seq) data from 6 patients with PD and 5 controls collected postmortem from the midbrain (Fig. S1A to C). We analyzed and counted the number of genes expressed in each cell, resulting in data from 41,427 cells and 19,832 gene features. Then, we mapped single-nucleus transcriptomes through uniform manifold approximation and projection (UMAP). According to the previously well-identified marker genes and machine learning cross-validation of cell type annotation, the cells were divided into the following 10 cell types: astrocytes, cholinergic neurons, dopaminergic neurons (DaNs), endothelial cells, ependymal cells, γ-aminobutyric acidergic (GABAergic) neurons, microglia, oligodendrocytes, oligodendrocyte precursor cells (OPCs), and pericytes (Fig. 1A). Next, to investigate the molecular differences between patients with PD and healthy controls (HCs), we validated the top 10 differentially expressed genes (DEGs; specifically, the top 10 downregulated DEGs) in patients with PD; *CDK5* was identified as the most substantially downregulated DEG in patients with PD (Fig. 1B). Specifically, this gene was markedly decreased in midbrain cholinergic neurons in patients with PD (Fig. 1C), the neurons that were filtered by the markers acetylcholinesterase (*ACHE*) and choline acetyltransferase (*CHAT*) (Fig. S1D) through model-based analysis of single-cell transcriptomics. We also found a decreased protein level of CDK5 (Fig. 1D and E) in the serum of patients with PD (Table S1).

### Mice with cholinergic-neuron-specific Cdk5 deficiency exhibit tremor and motor dysfunction

**Fig. 2.**
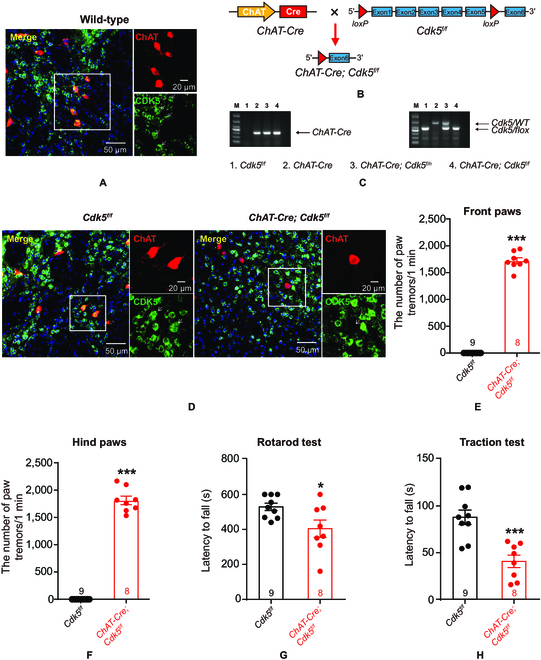
Mice with cholinergic-neuron-specific *Cdk5* deficiency exhibit tremor and motor dysfunction. (A) Representative image of CDK5 and ChAT in wild-type mice. The slices were stained with an anti-CDK5 antibody (green), anti-ChAT antibody (red), and 4′,6-diamidino-2-phenylindole (blue; marker for nuclei). Scale bar, 50 μm; magnified image, 20 μm. (B) The strategy used to generate cholinergic-neuron-specific *Cdk5*-cKO mice (*ChAT-Cre*;*Cdk5^f/f^* mice). Floxed *Cdk5* transgenic mice (*Cdk5^f/f^*) were crossed with *ChAT-Cre* mice. (C) Genotyping of *ChAT-Cre*;*Cdk5^f/f^* mice. *ChAT-Cre* primers generated a 300-bp product, and *Cdk5* primers generated a 460-bp product of the loxp-flanked allele or a 660-bp product of the wild-type allele. (D) Representative images of CDK5 and ChAT in *Cdk5^f/f^* mice and *ChAT-Cre*;*Cdk5^f/f^* mice. Scale bars, 50 μm; magnified images, 20 μm. (E and F) The number of paw tremors in *ChAT-Cre*;*Cdk5^f/f^* mice (*n* = 8) and *Cdk5^f/f^* mice (*n* = 9). Paw tremors in *ChAT-Cre*;*Cdk5^f/f^* mice were recorded for 1 min. ^***^P < 0.001, unpaired 2-tailed Student’s *t* test. Data represent the means ± SEM. (G and H) Quantification of the time spent in the rotarod (G) and traction (H) tests. *Cdk5^f/f^* mice (*n* = 9) and *ChAT-Cre*;*Cdk5^f/f^* mice (*n* = 8). ^*^P < 0.05 and ^***^P < 0.001, unpaired 2-tailed Student’s *t* test. Data represent the means ± SEM.

To examine the role of CDK5 in cholinergic neurons, we first confirmed CDK5 expression in cholinergic neurons through immunostaining. The data showed that CDK5 colocalized with choline acetyltransferase (ChAT)-positive cholinergic neurons (Fig. 2A), which suggested that CDK5 was enriched in cholinergic neurons. Then, we crossed *Cdk5^f/f^* mice, in which exon 1 to exon 5 of *Cdk5* were flanked by *loxP* sites, with *ChAT-Cre* mice to generate cholinergic-neuron-specific *Cdk5*-cKO mice (Fig. 2B and C). The immunostaining results indicated that ChAT-positive neurons did not express CDK5 in *ChAT-Cre*;*Cdk5^f/f^* mice (Fig. 2D). In addition, cholinergic-neuron-specific *Cdk5* deletion had no effect on body weight, brain weight, or the organ index (Fig. S2A to C).

Cholinergic neurons are implicated in motor control and motor processes; thus, we first explored whether *Cdk5* loss affected motor behavior in *ChAT-Cre*;*Cdk5^f/f^* mice. Video data (Movie S1) showed that all *Cdk5*-deficient mice exhibited paw tremors (Fig. 2E and F). In the rotarod test, *ChAT-Cre*;*Cdk5^f/f^* mice showed a shorter latency to fall from the rotating rod than *Cdk5^f/f^* mice (Fig. 2G). A similar result was also seen in the traction test (Fig. 2H). However, gait analysis revealed no difference between *ChAT-Cre*;*Cdk5^f/f^* mice and *Cdk5^f/f^* mice (Fig. S2D). To exclude the influence of alterations in the function of limb skeletal muscles on motor ability, we performed limb electromyography (EMG) in anesthetized mice [23]. The EMG results indicated no impairment of electrical activity in the limbs (Fig. S2G and H). The grip strength test showed that the *Cdk5* deficiency in cholinergic neuron weakens grip strength (Fig. S2I and J). Moreover, our results revealed no difference in performance in the Y-maze test (Fig. S2K) or the open-field test (Fig. S2L) between the groups.

Together, these results confirm that CDK5 plays a role in basic cholinergic-neuron-mediated motor function.

### Enhanced intrinsic excitability of striatal cholinergic neurons in mice with cholinergic-neuron-specific Cdk5 deficiency

**Fig. 3.**
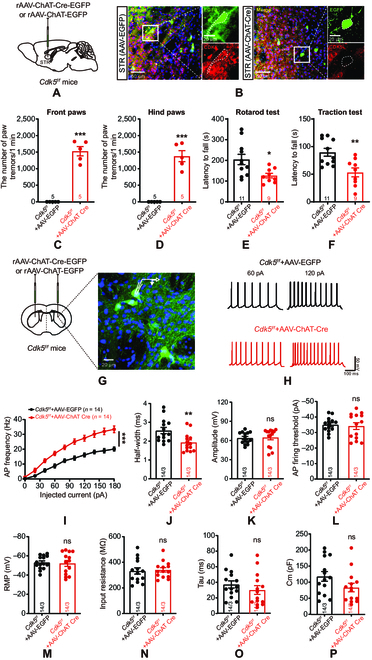
Elevation of intrinsic striatal cholinergic neuron excitability in mice with cholinergic-neuron-specific *Cdk5* deficiency. (A) Schematic diagrams of the injection of rAAV-ChAT-Cre-EGFP vectors or control vectors (rAAV-ChAT-EGFP) into the striatum of *Cdk5^f/f^* mice. (B) Representative confocal images to identify *Cdk5*-knockout efficiency in striatal cholinergic neurons. Scale bars, 50 μm; magnified images, 20 μm. (C and D) The number of paw tremors in *Cdk5^f/f^* mice injected with rAAV-ChAT-Cre-EGFP (*n* = 5) or rAAV-ChAT-EGFP (*n* = 5) vectors. Paw tremors were recorded for 1 min. ^***^P < 0.001, unpaired 2-tailed Student’s *t* test. Data represent the means ± SEM. (E and F) Quantification of the time spent in the rotarod (E) and traction (F) tests. *Cdk5^f/f^* mice injected with rAAV-ChAT-EGFP (*n* = 11) and rAAV-ChAT-Cre-EGFP (*n* = 9). ^*^P < 0.05 and ^**^P<0.01, unpaired 2-tailed Student’s *t* test. Data represent the means ± SEM. (G) Representative images of EGFP-positive neurons in the striata of *Cdk5^f/f^* mice injected with AAV vectors. Scale bar, 20 μm. (H and I) Representative APs and quantification of the AP frequency in EGFP-positive neurons in the striata of *Cdk5^f/f^* mice injected with rAAV-ChAT-Cre-EGFP (*n* = 14 cells from 3 mice) or rAAV-ChAT-EGFP (*n* = 14 cells from 3 mice) following current injections at 20 pA. ^***^P < 0.001, 2-way ANOVA followed by Tukey’s multiple comparisons test. Data represent the means ± SEM. (J) The AP half-width in cholinergic neurons in the striata of *Cdk5^f/f^* mice injected with rAAV-ChAT-EGFP (*n* = 14 cells from 3 mice) and rAAV-ChAT-Cre-EGFP (*n* = 14 cells from 3 mice). ^**^P < 0.01, unpaired 2-tailed Student’s *t* test. Data represent the means ± SEM. (K and L) Quantification of the AP amplitude and AP firing threshold in eGFP-positive neurons in the striata of *Cdk5^f/f^* mice injected with rAAV-ChAT-EGFP (*n* = 14 cells from 3 mice) and rAAV-ChAT-Cre-EGFP (*n* = 14 cells from 3 mice) under whole-cell recording. AP amplitude, *P* = 0.8376; AP firing threshold, *P* = 0.7283; unpaired 2-tailed Student’s *t* test. Data represent the means ± SEM. (M to P) Intrinsic membrane properties of striatal cholinergic neurons from *Cdk5^f/f^* mice injected with rAAV-ChAT-EGFP (*n* = 14 cells from 3 mice) and rAAV-ChAT-Cre-EGFP (*n* = 14 cells from 3 mice). Membrane potential, *P* = 0.7873; input resistance: *P* = 0.7818; Tau, *P* = 0.3343, *C*_m_: *P* = 0.0976; unpaired 2-tailed Student’s *t* test. Data represent the means ± SEM. STR, striatum; RMP, resting membrane potential; ns, not significant.

Striatal cholinergic neurons are involved in motor function, and, according to the Human Protein Atlas, *Cdk5* mRNA is enriched in the basal ganglia (Fig. S3A). Next, to validate the specific role of CDK5 in striatal cholinergic neurons in motor function, we administered a bilateral injection of adeno-associated virus (AAV) vectors carrying rAAV-ChAT-Cre-EGFP or rAAV-ChAT-EGFP (as the control) into the striatum of *Cdk5^f/f^* mice to conditionally knock out *Cdk5* in cholinergic neurons (Fig. 3A). In the striatum, the immunostaining data showed that the ratio of enhanced green fluorescent protein (EGFP)^+^-ChAT^+^ cells/ChAT^+^ cells was 88.94% ± 3.19%, and the ratio of EGFP^+^-ChAT^+^ cells/EGFP^+^ cells was 82.75% ± 2.42% (Fig. S3B and C), which indicated high infection efficiency. Twenty-one days after virus injection, the immunohistochemistry results indicated reduced expression of CDK5 in striatal cholinergic neurons in mice injected with rAAV-ChAT-Cre-EGFP (Fig. 3B). Behavioral analyses were performed to evaluate the motor function of striatal cholinergic *Cdk5*-deficient mice. The striatal cholinergic *Cdk5-*deficient mice exhibited static paw tremors in all 4 limbs compared with the control mice (Fig. 3C and D). During the 10-min rotarod test, the *Cdk5*-cKO mice exhibited a shorter latency to fall from the rotating rod than the control mice (Fig. 3E). Similarly, the *Cdk5*-cKO mice showed a shorter latency to fall from the metallic wire in the traction test (Fig. 3F). These results showed that the loss of *Cdk5* in striatal cholinergic neurons impaired the motor function of mice.

To further address the effect of *Cdk5* deficiency on the firing properties of striatal cholinergic neurons, we conducted electrophysiological recordings according to the whole-cell mode (Fig. 3G) [8]. *Cdk5* deficiency increased the frequency (Fig. 3H and I) and reduced the half-width (Fig. 3J) of action potentials (APs) in striatal cholinergic neurons in rAAV-ChAT-Cre-EGFP-injected mice compared with rAAV-ChAT-EGFP-injected mice. Other AP properties, including amplitude and firing threshold, did not differ between the 2 groups of mice (Fig. 3K and L). Moreover, no significant changes were found in the resting membrane potential, membrane time constant (Tau), input resistance (*R*_in_), or membrane capacitance (*C*_m_) between the 2 groups of mice (Fig. 3M to P).

We also performed electrophysiological recordings in striatal cholinergic neurons of *ChAT-Cre*;*Cdk5^f/f^* mice. To accurately identify cholinergic neurons, we generated *ChAT-Cre*;*Cdk5^f/f^*;*ChAT eGFP* mice. Consistent with striatal cholinergic neurons in *Cdk5-*cKO mice, the frequency of APs in cholinergic neurons was increased (Fig. S3D), and the AP half-width was reduced (Fig. S3E) in *ChAT-Cre*;*Cdk5^f/f^*;*ChAT eGFP* mice. Other AP properties (amplitude and threshold) were unaltered (Fig. S3F and G). Furthermore, cell-attached recordings indicated no change in the spontaneous firing rate of these neurons in *ChAT-Cre*;*Cdk5^f/f^*;*ChAT eGFP* mice (Fig. S3H and I). Whole-cell recordings showed no significant changes in the resting membrane potential, *R*_in_, Tau, or *C*_m_ (Fig. S3J to M). These data suggest that CDK5 mediates the electrophysiological activity of cholinergic neurons.

### Elevation of BK currents is associated with hyperexcitability of striatal cholinergic neurons in *ChAT-Cre;Cdk5^f/f^;ChAT eGFP* mice

**Fig. 4.**
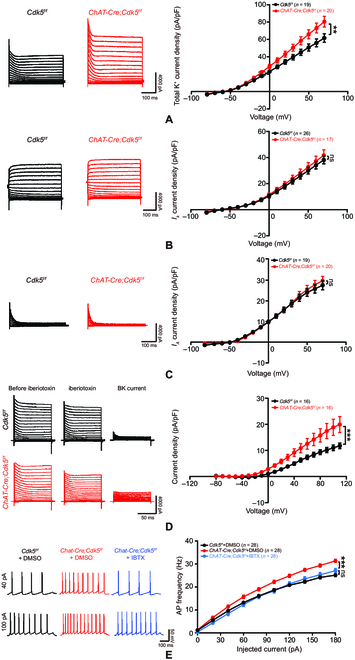
Increased BK currents are associated with hyperexcitability of striatal cholinergic neurons in *ChAT-Cre*;*Cdk5^f/f^*;*ChAT eGFP* mice. (A) Representative K^+^ currents and related summary data for striatal cholinergic neurons from *ChAT-Cre*;*Cdk5^f/f^*;*ChAT eGFP* mice (*n* = 20 cells from 4 mice) and *ChAT eGFP*;*Cdk5^f/f^* mice (*n* = 19 cells from 4 mice). ^**^P < 0.01, 2-way ANOVA followed by Tukey’s multiple comparisons test. Data represent the means ± SEM. (B) Representative delayed rectifier K^+^ currents (*I_K_* currents) and related summary data for striatal cholinergic neurons from *ChAT-Cre*;*Cdk5^f/f^*;*ChAT eGFP* mice (*n* = 17 cells from 2 mice) and *ChAT eGFP;Cdk5^f/f^* mice (*n* = 26 cells from 3 mice). *P* = 0.9991, 2-way ANOVA followed by Tukey’s multiple comparisons test. Data represent the means ± SEM. (C) Representative transient outward K^+^ currents (*I_A_* currents) and related summary data for striatal cholinergic neurons from *ChAT-Cre*;*Cdk5^f/f^*;*ChAT eGFP* mice (*n* = 20 cells from 4 mice) and *ChAT eGFP;Cdk5^f/f^* mice (*n* = 19 cells from 4 mice). *P* = 0.9986, 2-way ANOVA followed by Tukey’s multiple comparisons test. Data represent the means ± SEM. (D) Representative BK currents (acquired by subtracting currents recorded after the application of IBTX from currents before the application of IBTX) and related summary data for striatal cholinergic neurons from *ChAT-Cre*;*Cdk5^f/f^*;*ChAT eGFP* mice (*n* = 16 cells from 4 mice) and *ChAT eGFP*;*Cdk5^f/f^* mice (*n* = 16 cells from 4 mice). ^***^P < 0.001, 2-way ANOVA followed by Tukey’s multiple comparisons test. Data represent the means ± SEM. (E) Representative APs and quantification of the AP frequency of eGFP-positive neurons in the striata of *ChAT-Cre*;*Cdk5^f/f^*;*ChAT eGFP* mice (*n* = 28 cells from 5 mice) and *ChAT eGFP*;*Cdk5^f/f^* mice (*n* = 28 cells from 5 mice) with or without the application of IBTX following current injections at 20 pA from 0 to 180 pA. ^***^P < 0.001, 2-way ANOVA followed by Tukey’s multiple comparisons test. Data represent the means ± SEM. DMSO, dimethyl sulfoxide.

Voltage-gated Na^+^ channels and K^+^ channels are associated with the generation of APs and determine AP properties [24]. To test whether CDK5 alters Na^+^ channels and K^+^ channels, resulting in the hyperactivity of cholinergic neurons, we recorded Na^+^ and K^+^ currents under voltage-clamp conditions. We observed an increased density of K^+^ currents (Fig. 4A) and no change in the density of Na^+^ currents (Fig. S4A). There are numerous K^+^ currents including the following 3 main types: transient outward K^+^ currents (*I_A_* currents), delayed rectifier K^+^ currents (*I_k_* currents), and calcium-activated K^+^ currents (BK currents) [25]. We observed no change in the density of *I_k_* currents (Fig. 4B) or *I_A_* currents (Fig. 4C). However, BK channels were previously reported to regulate AP half-width; blockage of BK channels increased the AP half-width [26,27]. We used iberiotoxin (IBTX), a highly specific BK channel blocker, to isolate BK currents [28]. As shown in Fig. 4D, BK current density was increased in *ChAT-Cre;Cdk5^f/f^;ChAT eGFP* mice. More importantly, the hyperactivity of cholinergic neurons in *ChAT-Cre*;*Cdk5^f/f^*;*ChAT eGFP* mice was reversed by IBTX (Fig. 4E); thus, BK channels may induce the alteration in cholinergic neuron activity. In addition, CDK5 has been reported to regulate voltage-gated Ca^2+^ channels [29]. We therefore recorded Ca^2+^ currents in striatal cholinergic neurons under voltage-clamp conditions. However, we found no difference in the Ca^2+^ current density between groups in mice (Fig. S4B and C). These electrophysiology data demonstrate that increased BK currents due to *Cdk5* deficiency led to hyperexcitability of striatal cholinergic neurons.

### CDK5 negatively regulates BK channel activity via phosphorylation of T908

**Fig. 5.**
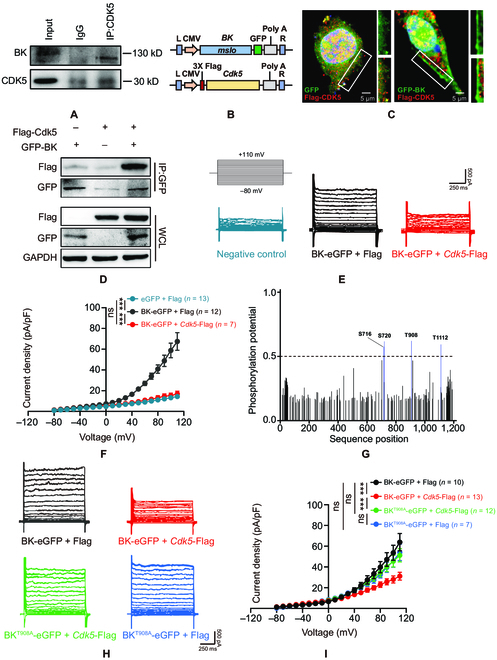
CDK5 negatively regulates BK channel activity via phosphorylation of T908. (A) Proteins pulled down with an anti-CDK5 antibody in the striatum were probed with an anti-BK channel antibody. (B) Schematic diagrams of the BK-channel-encoding *mslo* and *Cdk5* plasmids. (C) Representative images of the localization of CDK5 (red) and BK channels (GFP; green) in N2A cells after transfection with *BK* and *Cdk5* plasmids. Scale bars, 5 μm. (D) Proteins pulled down with an anti-eGFP antibody in N2A cells after 3×Flag-labeled *Cdk5* and *BK*-eGFP cotransfection were probed with an anti-Flag antibody. WCL, whole-cell lysate. (E) Representative BK current traces from N2A cells under voltage-clamp recording (10-mV increment). N2A cells were cotransfected with *BK*-eGFP and Flag (*BK*-eGFP + Flag) or *BK*-eGFP and *Cdk5*-Flag (*BK*-eGFP + *Cdk5*-Flag). (F) The current density of BK currents in N2A cells transfected with or without *Cdk5* plasmids. eGFP + Flag (*n* = 13), *BK*-eGFP + Flag (*n* = 12), and *BK*-eGFP + *Cdk5*-Flag (*n* = 7). ^***^P < 0.001, 2-way ANOVA followed by Tukey’s multiple comparisons test. Data represent the means ± SEM. (G) CDK5 phosphorylated sites on BK channel proteins were predicted with NetPhos 3.1. (H) Representative BK current traces under voltage-clamp recording (10-mV increment) from N2A cells cotransfected with *BK*-eGFP or *BK^T908A^*-eGFP and *Cdk5*-Flag or Flag. (I) Current–voltage relationships of BK currents for (H). *BK*-eGFP + Flag (*n* = 10), *BK*-eGFP + *Cdk5*-Flag (*n* = 13), *BK^T908A^*-eGFP + Flag (*n* = 7), and *BK^T908A^*-eGFP + *Cdk5*-Flag (*n* = 12). ^***^P<0.001, 2-way ANOVA followed by Tukey’s multiple comparisons test. Data represent the means ± SEM. IgG, immunoglobulin G; GAPDH, glyceraldehyde-3-phosphate dehydrogenase.

To explore how *Cdk5* deficiency contributes to the elevation of the BK-channel-mediated current density, we examined the association between CDK5 and BK channels. Coimmunoprecipitation and Western blotting data suggested that CDK5 binds directly to the BK channel in striatal cholinergic neurons (Fig. 5A). To further determine the role of interaction between CDK5 and BK channels, we constructed *mslo* (encoding BK)-eGFP (*BK*-eGFP) and *Cdk5*-Flag plasmids and transiently transfected them into Neuro 2A (N2A) cells (Fig. 5B). Confocal microscopy indicated that CDK5 and BK channels were colocalized in the N2A cell membrane (Fig. 5C). Similarly, coimmunoprecipitation data indicated that CDK5 binds directly to BK channels (Fig. 5D). Moreover, whole-cell patch-clamp recording suggested null N2A cells with a small current increase. These currents were up to 6-fold larger in *BK*-eGFP-transfected N2A cells than in null N2A cells and were strongly inhibited in *BK*-eGFP- and *Cdk5*-Flag-cotransfected N2A cells (Fig. 5E and F).

BK channels have multiple phosphorylation patterns [30]. Phosphorylation of serine-695 by protein kinase C blocks BK channel activity, and phosphorylation of serine-1151 by protein kinase C enhances the BK channel response to activation by guanosine 3′,5′-monophosphate-dependent protein kinase [31]. For BK channel activation, adenosine 3′,5′-monophosphate (cAMP)-dependent protein kinase must phosphorylate serine-899 in the α subunit of BK channels [32]. We used NetPhos 3.1 to predict the site of the BK channel protein targeted by CDK5 (scores greater than 0.5 were considered valid) [33] and identified 4 potential sites, i.e., serine-716 (S716), serine-720 (S720), threonine-908 (T908), and threonine-1112 (T1122) (Fig. 5G). To determine the site that CDK5 targets, we constructed BK channel mutants (*BK^S716A^*-eGFP, *BK^S720A^*-eGFP, *BK^T908A^*-eGFP, or *BK^T1112A^*-eGFP), cotransfected them with or without the *Cdk5*-Flag plasmid into N2A cells, and performed electrophysiology recordings. As expected, the increase in the BK current density induced by *BK*-eGFP transfection was blocked by cotransfection of *BK*-eGFP and *Cdk5*-Flag in N2A cells. More interestingly, there was no difference in the BK current density among N2A cells transfected with *BK*-eGFP and Flag, *BK^T908A^*-eGFP and Flag, or *BK^T908A^*-eGFP and *Cdk5*-Flag (Fig. 5H and I). However, the BK current density was decreased in N2A cells cotransfected with *Cdk5*-Flag, *BK^S716A^*-eGFP, *BK^S720A^*-eGFP, or *BK^T1112A^*-eGFP (Fig. S5A). Moreover, the BK current density did not differ among N2A cells transfected with *BK^S716A^*-eGFP, *BK^S720A^*-eGFP, or *BK^T1112A^*-eGFP compared to those transfected with *BK*-eGFP (Fig. S5B). These data indicate that CDK5 negatively regulates BK currents via phosphorylation of T908.

### Restoration of CDK5 expression in striatal cholinergic neurons ameliorates motor dysfunction

**Fig. 6.**
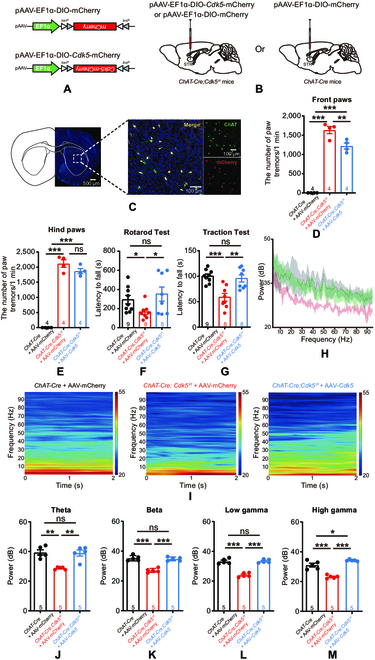
Restoration of CDK5 expression in striatal cholinergic neurons improves the motor function of *ChAT-Cre*;*Cdk5^f/f^* mice. (A) Schematic diagrams of *ChAT-Cre*-dependent AAV vectors for the overexpression of *Cdk5*. (B) Schematic diagram of the injection of Cre-dependent AAV vectors into the bilateral striatum of mice. (C) Representative images of mCherry/ChAT-positive cells in the striata of *ChAT-Cre;Cdk5^f/f^* mice. ChAT (green), CDK5 (mCherry; red); Scale bar, 500 μm (left) and 100 μm (right). (D and E) The number of front paw tremors (D) and hind paw tremors (E). The number of paw tremors in *ChAT-Cre;Cdk5^f/f^* mice was recorded for 1 min. *ChAT-Cre* + AAV-mCherry mice (*n* = 4), *ChAT-Cre;Cdk5^f/f^* + AAV-mCherry mice (*n* = 4), and *ChAT-Cre*;*Cdk5^f/f^* + AAV-*Cdk5*-mCherry mice (*n* = 4). ^**^P < 0.01 and ^***^P < 0.001, unpaired 2-tailed Student’s *t* test. Data represent the means ± SEM. (F and G) Quantification of the time spent on the accelerating rotarod and in the traction test. *ChAT-Cre* + AAV-mCherry mice (*n* = 9), *ChAT-Cre*;*Cdk5^f/f^* + AAV-mCherry mice (*n* = 8), and *ChAT-Cre*;*Cdk5^f/f^* + AAV-*Cdk5*-mCherry mice (*n* = 8). ^*^P < 0.05, ^**^P < 0.01, and ^***^P < 0.001, one-way ANOVA followed by Tukey’s multiple comparisons test. Data represent the means ± SEM. (H) Relative LFP power values in the striata of *ChAT-Cre* + AAV-mCherry mice (gray; *n* = 5), *ChAT-Cre*;*Cdk5^f/f^* + AAV-mCherry mice (red; *n* = 5) and *ChAT-Cre*;*Cdk5^f/f^* + AAV-*Cdk5*-mCherry mice (green; *n* = 5) following 32-channel recording electrode implantation (shaded bands indicate the SEM). (I) Representative LFP spectrograms from *ChAT-Cre* + AAV-mCherry mice, *ChAT-Cre*;*Cdk5^f/f^* + AAV-mCherry mice, and *ChAT-Cre*;*Cdk5^f/f^* + AAV-*Cdk5*-mCherry mice. (J to M) Relative LFP power values for different frequencies in the striata of mice. Theta (4 to 12 Hz) (J), beta (13 to 35 Hz) (K), low-gamma (36 to 65 Hz) (L), and high-gamma (66 to 95 Hz) (M) power in the striatum. *ChAT-Cre* + AAV-mCherry mice (*n* = 5), *ChAT-Cre;Cdk5^f/f^* + AAV-mCherry mice (*n* = 5), and *ChAT-Cre*;*Cdk5^f/f^*+ AAV-*Cdk5*-mCherry mice (*n* = 5), ^*^P < 0.05, ^**^P < 0.01, and ^***^P < 0.001, one-way ANOVA followed by Tukey’s multiple comparisons test. Data represent the means ± SEM.

To examine whether restoring CDK5 expression would alleviate motor dysfunction in *ChAT-Cre;Cdk5^f/f^* mice, we bilaterally injected an AAV-expressing *Cdk5* or mCherry (pAAV-EF1a-DIO-*Cdk5*-mCherry or pAAV-EF1a-DIO-mCherry) into the striata of *ChAT-Cre*;*Cdk5^f/f^* mice and an AAV-expressing mCherry into the striata of *ChAT-Cre* mice (Fig. 6A and B). After injection, we observed a large population of mCherry-labeled ChAT-positive neurons (Fig. 6C), indicating that CDK5 was expressed specifically in cholinergic neurons. Subsequently, we performed motor-related behavioral tests. The front paw tremors of *ChAT-Cre*;*Cdk5^f/f^* mice were alleviated by injection of pAAV-EF1a-DIO-*Cdk5*-mCherry, but there was no change in hind paw tremors (Fig. 6D and E). Furthermore, the results of the rotarod and traction tests indicated that restoration of CDK5 expression improved motor performance (Fig. 6F and G). To explore the effect of restoring CDK5 expression on electrical activity in the striatum, we recorded local field potentials (LFPs) in the striata of AAV-injected *Cdk5^f/f^* and *ChAT-Cre*;*Cdk5^f/f^* mice. We recorded 0- to 100-Hz LFP oscillations in the striata of freely behaving mice. We found that theta (4 to 12 Hz), beta (13 to 35 Hz), low-gamma (36 to 65 Hz), and high-gamma (66 to 95 Hz) oscillations were decreased in the striata of *ChAT-Cre*;*Cdk5^f/f^* mice; these decreases were significantly reversed by *Cdk5* overexpression (Fig. 6H to M).

## Discussion

In the present study, we illustrated that the BK channel is a CDK5 target that plays essential roles in modulating the activity of striatal cholinergic neurons, which are implicated in motor control and motor processes. By elucidating the electrophysiological mechanisms underlying *Cdk5* deficiency-mediated motor dysfunction, we found hyperexcitability of striatal cholinergic neurons, reduced AP half-width, and increased BK current density according to whole-cell recordings in *ChAT-Cre*;*Cdk5^f/f^* mice. At the cellular level, further mechanistic data suggested that CDK5 phosphorylates BK at T908 and negatively regulates BK currents. In addition, we demonstrated that restoration of CDK5 expression in striatal cholinergic neurons ameliorated cholinergic-neuron *Cdk5*-deficit-triggered dyskinesia-like behaviors.

The cardinal dyskinesia symptoms in PD include static tremor, rigidity, and postural instability [34]. Abnormal activity of striatal cholinergic neurons has been identified to be essential to dyskinesia-like behaviors and motor symptoms of PD. The upregulation of regulator of G protein signaling 4 (RGS4) and the activation D5-cAMP-ERK1/2 signaling pathway are reported to mediate hyperexcitability of striatal cholinergic neurons [35,36]. Neuronal CDK5 is required for spatial learning and recurrent excitatory circuits in the hippocampus [37,38] and proper multipolar-to-bipolar transition in the cortex [39]. Here, we showed that *CDK5* mRNA decreased markedly in patients with PD. CDK5 was also reduced in the serum of patients with PD. Consistent with these findings, *Cdk5* deficiency in cholinergic neurons triggered dyskinesia-like behaviors. Striatal cholinergic neurons exhibit 2 periodic firing patterns, i.e., rhythmic bursts and single spikes, and spontaneous firing patterns are caused by K^+^-current-mediated afterhyperpolarization [40]. Here, *Cdk5* deficiency had no effect on the spontaneous firing rate in cell-attached voltage-clamp recordings. However, in whole-cell patch-clamp recordings, *Cdk5* deficiency increased the frequency and decreased the half-width of APs in striatal cholinergic neurons after current injections, which may have accounted for the dyskinesia-like behaviors observed in *ChAT-Cre*;*Cdk5^f/f^* mice. Hence, our model has the advantage of mimicking, at least in part, the aetiology and symptomatology of dyskinesia-like behaviors in neurological disorders.

How does the reduction in *Cdk5* signaling in striatal cholinergic neurons contribute to motor dysfunction? We examined the voltage-gated Na^+^ and K^+^ currents, which mediate the formation and process of APs [26], and BK currents, which contribute to repolarization [14]. Here, we found that voltage-gated Na^+^ and K^+^ currents were unchanged in *Cdk5*-deficient cholinergic neurons. BK channels have profound effects on neuronal excitability [41,42]. Numerous studies have indicated that activation of BK channels enhances neuronal excitability [43–45]. In the present study, we used IBTX to segregate BK currents in striatal cholinergic neurons; we found increased BK currents in *ChAT-Cre*;*Cdk5^f/f^* mice. Next, we identified T908 as the phosphorylation site of CDK5 on BK channels. These data suggest that BK channels, which link intracellular molecular signals and neuronal electrical signals [46], are negatively regulated by CDK5 via phosphorylation at T908. Thus, deficit of CDK5 in striatal cholinergic neurons increased BK currents, leading to cholinergic neuron hyperexcitability.

The therapeutic relevance of our findings is highlighted by the amelioration of dyskinesia-like behaviors after local restoration of CDK5 in *ChAT-Cre*;*Cdk5^f/f^* mice. Motor deficits are associated with the emergence of exaggerated β oscillations in PD [47,48]. Here, we show that striatal cholinergic CDK5 regulates motor function through modulating frequency bands (alpha, beta, and gamma) in the corticostriatal circuit. The therapeutic effects of restoring CDK5 on LFPs highlight the potential clinical applications of closed-loop neuromodulation. This application is consistent with the idea that local intervention at the level of the striatum, in both rodents and humans, ameliorates dyskinesia-like phenotypes [49–52]. In future, it would be interesting to further explore whether CDK5-induced phosphorylation of BK channels also involves in dyskinesia-like behaviors of other neurological diseases.

In conclusion, our study identified BK channels in striatal cholinergic neurons as new targets for CDK5 phosphorylation, and we described electrophysiological and molecular mechanisms of *Cdk5*-deficiency-mediated dyskinesia-like behaviors. The association between CDK5 and its neuron-specific activator molecules p35 and p39 is essential for kinase activation [53,54]. In contexts where high BK current densities in striatal cholinergic neurons are associated with hyperexcitability of cholinergic neurons, the novel CDK5 activator may provide an intervention target to suppress dyskinesia-like behaviors in neurological disorders.

## Materials and Methods

### Animals

All mice were housed in group and in ventilated cages under a 12-h light/dark schedule and a 22 ± 1 °C temperature to maintain conventional healthy states [55,56]. All experimental protocols in mice were strictly performed in compliance with the guide of Nanjing Medical University Animal Experimentation Committee.

*ChAT-Cre* mice (stock no. 006410, The Jackson Laboratory) were crossed with *Cdk5^f/f^* mice (stock no. 014156, The Jackson Laboratory) [57] to generate *ChAT-Cre;Cdk5^f/f^* mice. *ChAT^BAC^-eGFP* mice (EGFP was expressed in cholinergic neurons under the direction of choline acetyltransferase transcriptional regulatory elements; stock no. 007902, The Jackson Laboratory) were crossed with *Cdk5^f/f^* mice and *ChAT-Cre;Cdk5^f/f^* mice respectively for the electrophysiology recording of cholinergic neurons.

### Patient samples

Patients with PD with motor disorders were enrolled in this study. Serum samples were collected from age- and race-matched controls and patients. All patients and controls were 50 to 80 years old and Asian race. The average age was 67.2 years old for patients with PD and 67.2 years old for HCs. The study with patients is approved by the Ethics Committee of Affiliated Brain Hospital of Nanjing Medical University with number 2021-KY007-01.

### The snRNA-seq data analysis

Major cell types from the midbrain of patients with PD and HCs were identified and annotated by interrogating the expression patterns of known marker genes: cholinergic neurons (marked by *CHAT* and *ACHE*) and processing snRNA-seq data analysis as reported [58].

### Virus vectors

To overexpress *Cdk5* specifically in cholinergic neurons of striatum, the pAAV-EF1α-DIO*-Cdk5*-mCherry (1 × 10^12^ viral particles/ml) vector was constructed using *Cdk5* with a 3×Flag. The pAAV-EF1α-DIO-mCherry (1 × 10^12^ viral particles/ml) was used as control. The pAAV-EF1α-DIO-*Cdk5*-mCherry was bilaterally injected into the striatum of *ChAT-Cre;Cdk5^f/f^* mice. The pAAV-EF1α-DIO-mCherry was bilaterally injected into the striatum of *ChAT-Cre* and *ChAT-Cre;Cdk5^f/f^* mice. Both of AAV vectors were packaged into AAV2/9 serotype by OBiO Technology (Shanghai, China).

To eliminate the striatal cholinergic neuron CDK5, the rAAV-ChAT-Cre-EGFP (5.1 × 10^12^ viral particles/ml) or the rAAV-ChAT-EGFP (as control) (2.1 × 10^12^ viral particles/ml) was bilaterally injected into the striatum of *Cdk5^f/f^* mice. These AAV vectors were packaged into AAV2/9 serotype by BrainVTA (Wuhan, China).

### Stereotaxic injections

Mice were anesthetized with 1.5% to 2% isoflurane gas/oxygen mixture and fixed to the stereotaxic apparatus [24,59]. An AAV of 1.2 μl was bilaterally injected into the striatum (anterior–posterior, +0.8 mm from bregma; medial–lateral, ±2.0 mm; dorsal–ventral, −3.0 mm) using the stereotaxic frame at postnatal day 60 (P60). The injection speed was 80 nl/min. After the injection, the injector was retained for another 7 min and then slowly withdrawn. The behavioral experiments were conducted 3-week after injection.

### Behavioral assays

The mice used for behavioral assays are male at the age of 4 to 6 months; besides, the behavioral experiments on the mice injected with AAVs were performed at 2 to 3 months old.

Rotarod test was performed as previously reported [24]. Briefly, mice were trained to run on an accelerating rotarod for 10 min each day (day 1, 4 rpm/min; day 2, from 4 to 15 rpm/min gradually). After 2 d of training, the mice were tested at 4 to 40 rpm/min 3 times each. The latency of falling off the rod was recorded and averaged out.

Traction test was conducted as previously described [60] to estimate muscle strength and equilibrium. The test included 3 trials. Mice forelimbs were placed on a metallic wire (diameter, 2 mm). The latency of falling from the wire was recorded and averaged out. The maximal test duration was 120 s to avoid fatigue.

Y-maze test was used to examine the spatial working memory of mice. As described previously [59], 3 successive entries were defined as an alternation.

Open-field test was applied to reveal anxiety-like behavior and motor function in a plastic cube box (40 cm × 40 cm) [59]. All mouse movement were recorded by video and analyzed with VideoTrack v3 program (ViewPoint Life Sciences). Total distance of each mouse was calculated to assess general locomotor activity.

Footprint test was done as previously described [61]. Mice were placed in an enclosed walkway (150-cm length × 15-cm width × 30-cm height) on a glass plate and traversed 3 times from one side of the walkway to the other each day. After 2 d of training, the mice footprints were printed touching with the glass plate green light through a high-speed video camera. The time through the walkway, the stride length, and front/hind paw overlap of footprints were analyzed.

Grip strength test was conducted using the grip test system [62]. Mice were placed on a metal grip with 4 limbs or the front limbs and were pulled back by the tail gently until the limbs of mice were disconnected with the grip. Each mouse was tested 3 times. The average values were calculated.

### Brain slice preparation and electrophysiologic recording

Mice at the age of P21 to P30 were anesthetized. As described previously [59], the brain was immediately taken away and put into cold artificial cerebrospinal fluid, and then striatum slices were cut using vibratome (Leica VT 1200S). The slices were transferred into the normal artificial cerebrospinal fluid solution for electrophysiology recording [59].

Cell-attached recordings were applied to measure the spontaneous firing APs of cholinergic neurons as described before [63].

Whole-cell recordings were performed from striatum cholinergic neurons with a MultiClamp 700B amplifier as described previously [64]. Glass micropipettes (3 to 8 MΩ) were used to established tight seals at −60mV for cholinergic neurons. Under current-clamp patch, APs, input resistance (*R*_in_), membrane time constant (Tau), and membrane capacitance (*C*_m_) were recorded.

Na^+^ currents and K^+^ currents were recorded in voltage-clamp mode. For Na^+^ currents recording, the bath solution contained tetraethylammonium chloride (10 mM; Aladdin) and CsCl (0.1 mM; Aladdin), the Na^+^ in intracellular solution was replaced by Cs^+^. The total K^+^ currents were recorded in the presence of tetrodotoxin (1 μM; Tocris). *I_k_* currents were recorded at voltages of −120 and +10 mV for 200 ms, followed by −80- to +70-mV voltages, with 10-mV increments, and 1,2-bis(2-aminophenoxy)ethane-*N*,*N*,*N*′,*N*′-tetraacetic acid (1 mM; Sigma-Aldrich) was added to electrode internal fluid to inhibit calcium-dependent potassium currents [65]. *I_A_* currents were inactivated at voltages of −120 and +10 mV for 200 ms and then calculated by subtracting the remaining K^+^ currents from total K^+^ currents.

BK channel activity was recorded under voltage-clamp mode as previously reported [28]. Before and after the application of IBTX (150 nM; APExBIO), currents were respectively generated at voltages from −80 to +120 mV in 10-mV steps. The tetrodotoxin (1 μM) was also added to the bath solution to eliminate spontaneous activity. IBTX-sensitive BK currents were obtained by subtracting currents before the application of IBTX from currents after the application of IBTX.

Calcium currents were recorded according to previously used protocol [66].

Data were analyzed using MATLAB (MathWorks, Natick, MA, USA), Clampfit 10 (Molecular Devices), and MiniAnalysis software (Synaptosoft).

### Immunohistochemistry

The immunohistochemistry staining was performed as previously [24]. Briefly, 40-μm-thick brain slices were incubated with 0.1% Triton X-100 in phosphate-buffered saline and blocked with 3% bovine serum albumin in phosphate-buffered saline. Finally, the slices were incubated with primary antibodies as follows: anti-ChAT (Millipore; 1:500) and anti-CDK5 (Cell Signaling Technology; 1:200). Images were captured through a confocal laser scanning microscope (Zeiss LSM 800, Germany).

### Cell culture and plasmid

Cell culture and transfection were done as before [24]. N2A cells were obtained from American Type Culture Collection, the cells were cultured in Dulbecco’s modified Eagle’s medium with 10% fetal bovine serum, penicillin (100 U/ml), and streptomycin (100 μg/ml) at a 37°C incubator with 5% CO_2_. After cultured for 24 h, the N2A cells were transfected with plasmids using Lipofectamine 3000 (Invitrogen, Carlsbad, CA, USA) for 12 h and replaced with fresh medium for another 48-h culture.

The pcDNA3.1-3X-Flag-*Cdk5* was provided by X. Guo (Department of Neurobiology, Nanjing Medical University). Full-length *mslo* (encoding BK) (NM_001253378.1) cDNA was amplified from the cDNA of primary neuronal cells using the following primer set: sense, 5′- A​AA​CTC​GAG​AT​GGC​AAA​CGG​TGG​CGG​CGG​CGG​CGGCGGC-3′; antisense, 5′- AAAGCGGCCGCCACATTCATCTTCAACTTCTCTGAT-3′. In these primers, Xho I and Not I restriction site sequences have been underlined. The polymerase chain reaction products were cloned into the pEGFP-N1 vector. BK carrying the S716A mutation was amplified using the following primer set: sense, 5′-GGGCATGAGGAACGCGCCCAACACCTC-3′; antisense, 5′-GAGGTGTTGGGCGCGTTCCTCATGCCC-3′. BK carrying the S720A mutation was amplified using the following primer set: sense, 5′-CTCGCCCAACACCGCCCCGAAGCAGAT-3′; antisense, 5′-ATCAGCTTCGGGGCGGTGTTGGGCGAG-3′. BK carrying the T908A mutation was amplified using the following primer set: sense, 5​′-C​TAA​TTC​CCA​AGG​ATT​CGC​ACC​TCC​TGG​AATGGAC-3′; antisense, 5​′-G​TCC​ATT​CCA​GGC​GGT​GCG​AAT​CCT​TGG​GAATTAG-3′. BK carrying the T1112A mutation was amplified using the following primer set: sense, 5′-CCCACCTCAGCGCCCCCAGCCAG-3′; antisense, 5′-CTGGCTGGGGGCGTGAGGTGGG-3′. All constructions were ensured by sequencing.

### Immunoprecipitation and Western blot

Immunoprecipitation (IP) and Western blot (WB) were conducted as previously described [24]. Briefly, after transfection, N2A cell lysates were precleared with immunoglobulin G-Agarose beads (Beyotime) for 2 h and incubated supernatant with anti-CDK5 affinity gel beads, or anti-GFP affinity gel beads for overnight at 4 °C on a gentle shaker. Immunoprecipitated samples were washed with lysis buffer and eluted with 2× SDS loading buffer and then subjected to WB analysis. Primary antibodies were used as follows: anti-GFP antibody (Abcam; 1:100 for IP and 1:2,000 for WB), anti-CDK5 (Santa Cruz Biotechnology; 1:50 for IP and 1:1,000 for WB), anti-BK (Neuromab; 1:1,000), anti-Flag (Sigma-Aldrich; 1:5,000), and anti-glyceraldehyde-3-phosphate dehydrogenase (Cell Signaling Technology; 1:5,000).

### EMG analysis

The EMG of leg muscles were recorded as described before [67]. The gastrocnemius muscles of mice left hind limbs were exposed via a left hind limb incision. The gastrocnemius muscle was then hung on a pair of paralleled silver electrodes. The spontaneous muscle electrical activity was recorded and amplified with an AD/CD differential amplifier (model DP-304, Warner Instruments, Hamden, CT) [23]. The signals between 100 and 3,000 Hz were filtered and saved for further analysis.

### Surgery for implantation of tetrodes

Mice were deeply anesthetized with 1.5% to 2% isoflurane gas/oxygen mixture and fixed on the stereotactic apparatus [56,68]. Then, a hole was drilled over the right arcuate nucleus for implantation (striatum from bregma: anterior–posterior, +0.8 mm; medial-lateral, 0.2 mm). For LFP recording, the tetrodes were implanted into the arc with an average depth of 3 mm to reach the striatum. After tetrodes implantation, the signals were amplified by a 32-channel amplifier and sampled at 20 kHz by a Neuralynx recording system.

### LFP recordings

For LFP recordings, signals were amplified (1,000 × gain; Neuralynx), filtered at 0.1 to 250 Hz, and sampled at 1 kHz. The analysis of the LFP signals was conducted by a program written in MATLAB.

### Spectral analysis and phase–amplitude coupling

Spectral power was obtained by using MATLAB’s wavelet method (MathWorks), continuous wavelet transform, and Morlet wavelets [69]. Power spectral analysis of different oscillations was performed via the multitaper method in the MATLAB.

### Statistical analysis

Data were analyzed by experimenters blinded to the mice genotype. GraphPad Prism 8 was applied for statistical analysis. All data were displayed as means ± SEM. Unpaired 2-tailed Student’s *t* test was performed for 2 independent group comparisons. One-way analysis of analysis of variance (ANOVA) followed by Tukey’s post hoc test was used for multiple normally distributed data. Two-way ANOVA was used for dataset with 2 factors, followed by Bonferroni’s multiple comparison test. ^*^P < 0.05 was considered to be statistically significant.

## Data Availability

All data required to support the conclusions are presented in the main text and the Supplementary Materials.
