# Supplementary material for: Striatal CDK5 Regulates Cholinergic Neuron Activation and Dyskinesia-like Behaviors through BK Channels
Source: Research (Wash D C). 2023 Apr 18;6:0121. doi: 10.34133/research.0121 (PMC10202382; doi:10.34133/research.0121)
Supplement: Supplementary 1 — Table S1 Figs. S1 to S5 [file research.0121.f1.docx]

**Supplemental Materials**

**Striatal CDK5** **Regulates Cholinergic Neuron Activation and** **Dyskinesia-like Behaviours through BK Channels**

Chu Tong^1†^, Peng-Xiang Min^2†^, Qian Zhang^1^, Ru-Xin Gu^3^, Yao-Hai Wen^1^, Yi Shi^1^, Yu-Huan Bao^2,4^, Xiang Chen^2^, Yi-Xuan Zhang^2,5^, Xing-Feng Mao^1,2^, Hao-Yang Yuan^1^, Xiu-Xiu Liu^2^, Takuya Sasaki^6^, Li Zhang^3,7*^, Feng Han^2, 7*^, Ying-Mei Lu^1,7*^

**Affiliations**

*^1^Department of Physiology, School of Basic Medical Sciences, Nanjing Medical University, Nanjing 211166, China.*

*^2^International Joint Laboratory for Drug Target of Critical Illnesses, School of Pharmacy, Nanjing Medical University, Nanjing 211166, China.*

*^3^Department of Geriatrics, Nanjing Brain Hospital Affiliated to Nanjing Medical University, Nanjing, 210029, China.*

*^4^Institute of Pharmacology and Toxicology, College of Pharmaceutical Sciences, Zhejiang University, Hangzhou 310058, China.*

*^5^Gusu School, Nanjing Medical University, Suzhou Municipal Hospital, The Affiliated Suzhou Hospital of Nanjing Medical University, Suzhou, 215002, China.*

*^6^Department of Pharmacology, Graduate School of Pharmaceutical Sciences, Tohoku University, Sendai 980-8578, Japan.*

*^7^Institute of Brain Science, the Affiliated Brain Hospital of Nanjing Medical University, Nanjing 211166, China.*

^†^These authors contributed equally to this work.

^*^Correspondence should be addressed to Yingmei Lu; lufx@njmu.edu.cn, Feng Han; fenghan169@njmu.edu.cn and Li Zhang; dr_linda@njmu.edu.cn.

**Supplementary Information**

**Supplementary Table 1** Case details of PD patients with dyskinesia-like behaviors.

| Disorder | Sex | Age (Years) | Race | Disease stage | Disease duration  (Years) | Treatment with L-dopa | Usage time of L-dopa |
| --- | --- | --- | --- | --- | --- | --- | --- |
| PD-1 | F | 64 | Asian | Stage two | 15 | Yes | 9 days |
| PD-2 | F | 72 | Asian | Stage three | 7 | Yes | 6 days |
| PD-3 | F | 55 | Asian | Stage two | 10 | Yes | 10 years |
| PD-4 | M | 69 | Asian | Stage one | 5 | Yes | 9 days |
| PD-5 | F | 72 | Asian | Stage three | 18 | No, with severe adverse drug reaction | N/A |
| PD-6 | F | 71 | Asian | Stage one | 5 | Yes | 5 years |
| Control-1 | F | 75 | Asian | N/A | N/A | N/A | N/A |
| Control-2 | F | 53 | Asian | N/A | N/A | N/A | N/A |
| Control-3 | F | 80 | Asian | N/A | N/A | N/A | N/A |
| Control-4 | M | 75 | Asian | N/A | N/A | N/A | N/A |
| Control-5 | F | 70 | Asian | N/A | N/A | N/A | N/A |
| Control-6 | F | 50 | Asian | N/A | N/A | N/A | N/A |

**Supplementary Figures**

**
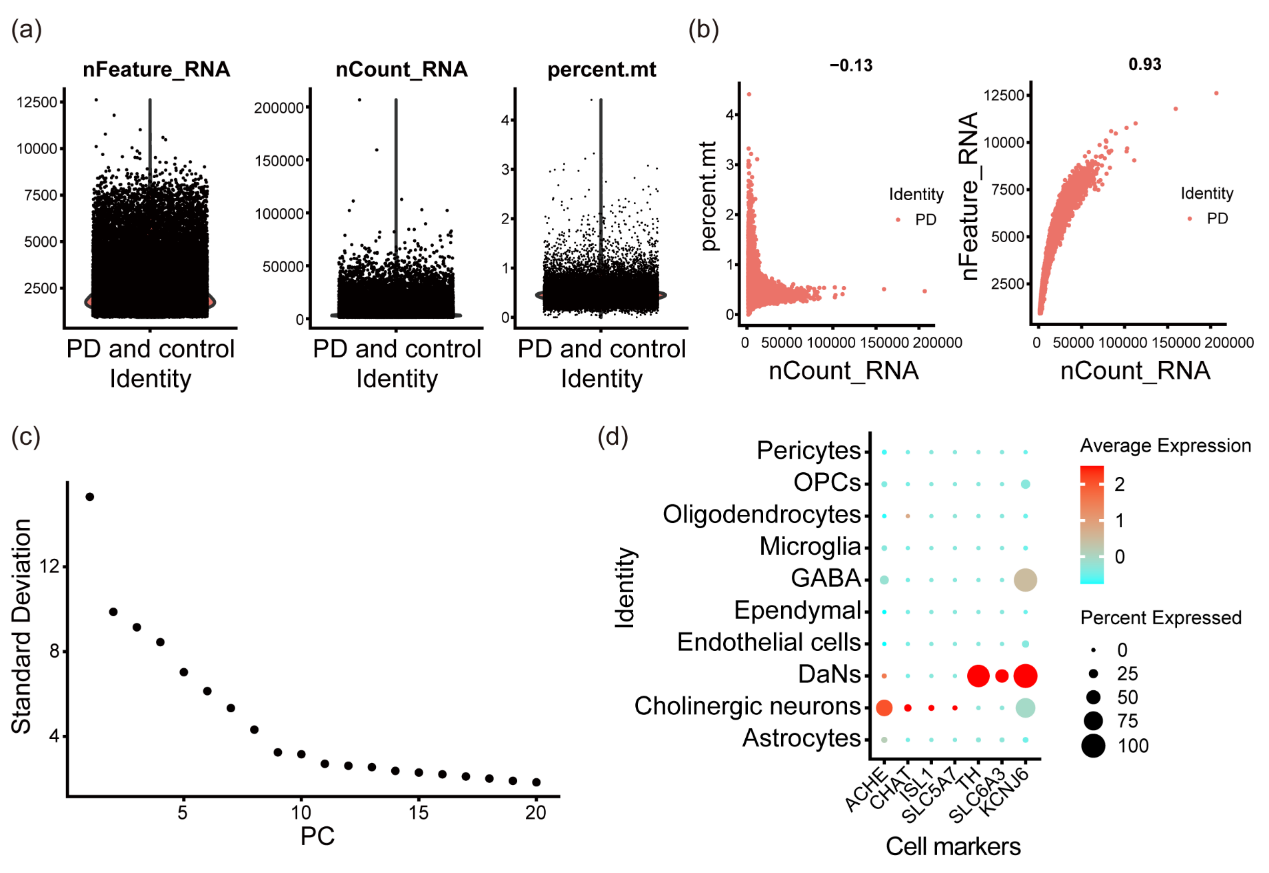
**

**FIGURE S1: snRNA-seq data analysis of quality control (QC) and data dimensionality reduction, related to FIGURE. 1.** (a) The QC metrics of the dataset. nFeature_RNA indicated the number of unique genes detected in each single cell. nCount_RNA represented the number of all molecules detected within a cell. Percent.mt showed the percentage of reads that map to the mitochondrial genome. (b) The nCount_RNA-percent.mt FeatureScatter and nCount_RNA-nFeature_RNA FeatureScatter were plotted to visualized relationships between two features. (c) The elbow plot ranked principle components (PC) based on the percentage of variance. (d) Dotplot of cholinergic neurons representative marker genes (*ACHE*, *CHAT*, *ISL1*, and *SLC5A7*) expression in different cell types.

**
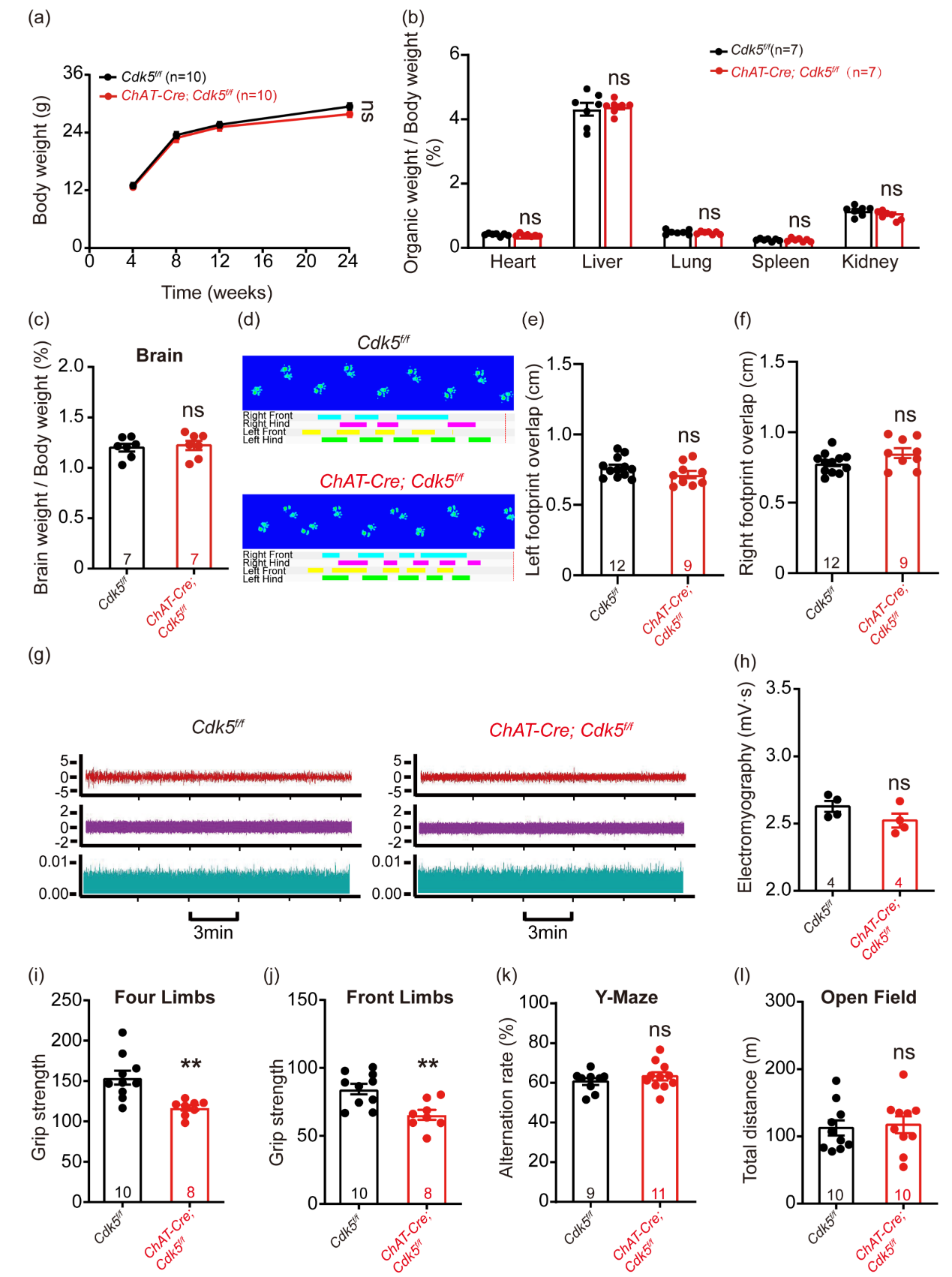
**

**FIGURE S2: *Cdk5* deficit had no effect on basal parameters of *ChAT-Cre;Cdk5^f/f^* mice, related to FIGURE. 2.** (a) Body weights of *Cdk5^f/f^* mice (*n* = 10) and *ChAT-Cre;Cdk5^f/f^* mice (*n* = 10). *p*=0.6108, Two-way ANOVA followed by Tukey’s multiple comparisons test. Data represents mean ± SEM. (b) Major organ weight compared with body weight. heart, *P*=0.4030; liver, *P*=0.7293; lung, *P*=0.5094; spleen, *P*=0.8089; kidney, *P*=0.2754, unpaired two-tailed Student’s *t* test. Data represents mean ± SEM. (c) Brain weights of *Cdk5^f/f^* mice (*n* = 7) and *ChAT-Cre;Cdk5^f/f^* mice (*n* = 7). *P*=0.7206, unpaired two-tailed Student’s *t* test. Data represents mean ± SEM. (d) Representative paw prints of *ChAT-Cre;Cdk5^f/f^* and *Cdk5^f/f^* mice captured by a gait instrument. (e) and (f) *ChAT-Cre;Cdk5^f/f^* (*n* = 9) and *Cdk5^f/f^* mice (*n* = 12)showed similar extent of left and right footprint overlap. Left footprint overlap, *P*=0.1516; right footprint overlap, *P*=0.0783, unpaired two-tailed Student’s *t* test. Data represents mean ± SEM. (g) Representative hind limbs electromyography of *ChAT-Cre;Cdk5^f/f^* and *Cdk5^f/f^* mice for 15 minutes. (h) Summary of 40 minutes’ hind limbs electromyography results indicated intact electrical activity at peripheral neuromuscular junctions. n=4 mice per group, *P*=0.16, unpaired two-tailed Student’s *t* test. Data represents mean ± SEM. (i,j) The grip strength of four limbs (i) and front limbs (j) in *Cdk5^f/f^* mice (n=10) and *ChAT-Cre;Cdk5^f/f^* mice (n=8). ^**^*P*<0.01, unpaired two-tailed Student’s *t* test. Data represent the mean ± SEM. (k) *ChAT-Cre;Cdk5^f/f^* mice exhibited intact cognitive function in the Y-Maze test. *Cdk5^f/f^* mice (*n* = 9), and *ChAT-Cre;Cdk5^f/f^* mice (*n* = 11). *P*=0.36, unpaired two-tailed Student’s *t* test. Data represents mean ± SEM. (l) The total traveled distance of *ChAT-Cre;Cdk5^f/f^* mice and *Cdk5^f/f^* mice. n=10 mice per group, *P*=0.78, unpaired two-tailed Student’s *t* test. Data represents mean ± SEM.


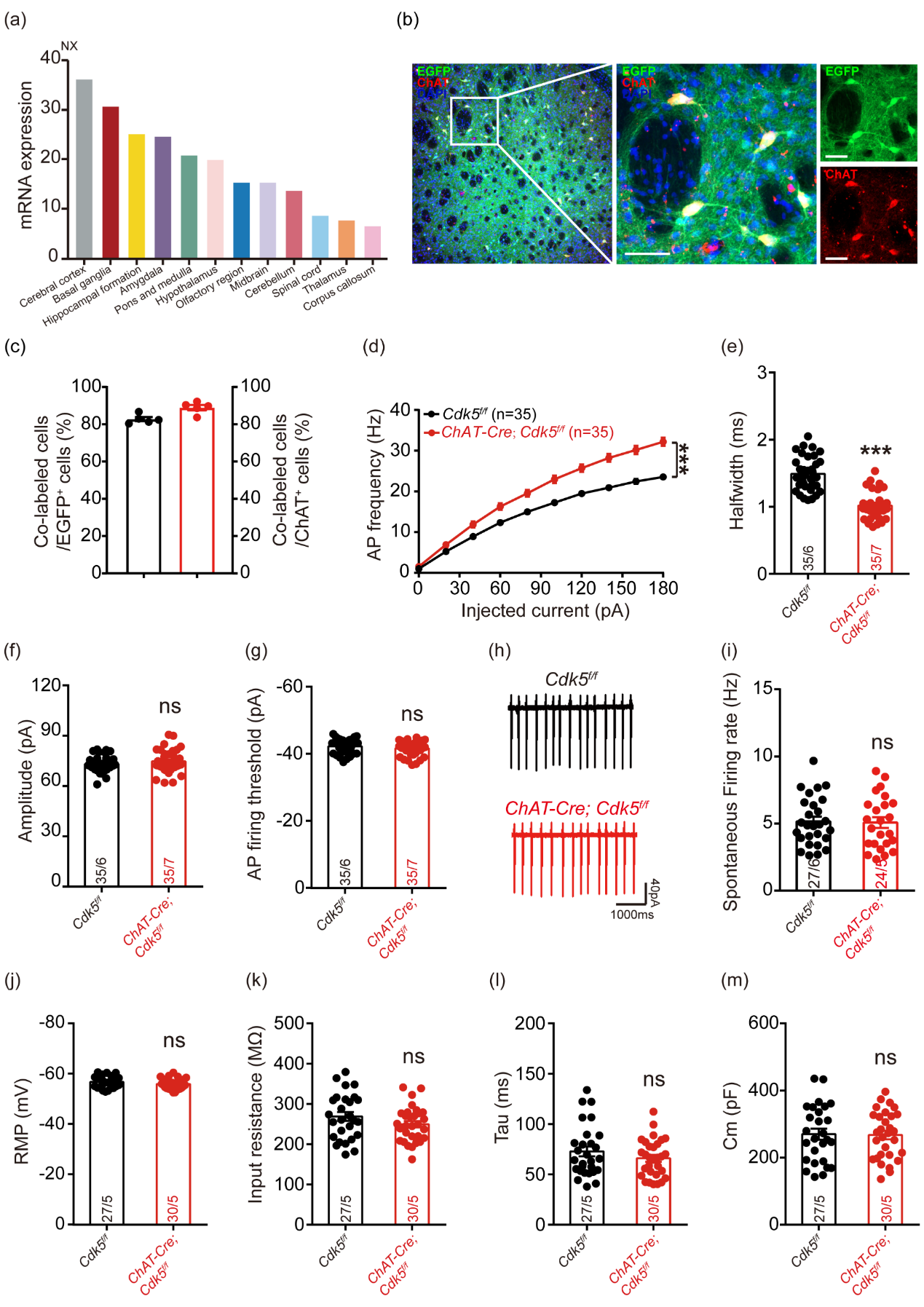


**FIGURE S3: Cholinergic neuron-specific *Cdk5* deficiency elevates intrinsic striatal Cholinergic neurons excitability under whole-cell patch-clamp recordings, related to FIGURE. 3.** (a) mRNA expression of cerebral *Cdk5* from The Human Protein Atlas. (b) Representative images of EGFP (green) and ChAT (red) in striatum of mice injected with rAAV-ChAT-Cre-EGFP. Scale bar = 100 μm; magnified images = 50 μm. (c) The percentage of EGFP^+^ and ChAT^+^ co-labeled cells relative to EGFP^+^ cells (left) or ChAT^+^ cells (right) in striatum. n=5 mice. (d) Quantification of the AP frequency in eGFP-positive neurons in the striata of *ChAT-Cre*;*Cdk5^f/f^*;*ChAT eGFP* mice (*n* = 35 cells from 7 mice) and *ChAT eGFP*;*Cdk5^f/f^* mice (*n* = 35 cells from 6 mice) at P21-P30 following current injections at 20 pA. *^***^P*<0.001, two-way ANOVA followed by Tukey’s multiple comparisons test. Data represents mean ± SEM. (e) The halfwidth of APs in cholinergic neurons in the striata of *ChAT-Cre*;*Cdk5^f/f^*;*ChAT eGFP* mice (*n* = 35 cells from 7 mice) and *ChAT eGFP*;*Cdk5^f/f^* mice (*n* = 35 cells from 6 mice). Two-way ANOVA followed by Tukey’s multiple comparisons test. Data represents mean ± SEM. (f) and (g) Quantification of the AP properties of eGFP-positive neurons in the striata of *ChAT-Cre*;*Cdk5^f/f^*;*ChAT eGFP* mice (*n* = 35 cells from 7 mice) and *ChAT eGFP*;*Cdk5^f/f^* mice (*n* = 35 cells from 6 mice). Amplitude: *P*=0.2689, AP firing threshold: *P*=0.2612, unpaired two-tailed Student’s *t* test. Data represents mean ± SEM. (h) Representative spontaneous firing of striatal cholinergic neurons acquired by cell-attached voltage clamp recordings. (i) Summary of spontaneous firing rate. *ChAT eGFP*;*Cdk5^f/f^* mice (*n* = 24 cells from 5 mice) and *ChAT-Cre*;*Cdk5^f/f^*;*ChAT eGFP* mice (*n*=27 cells from 6 mice). *P*=0.87, unpaired two-tailed Student’s *t* test. Data represents mean ± SEM. (j-m) Intrinsic membrane properties of striatal cholinergic neurons from *ChAT-Cre*;*Cdk5^f/f^*;*ChAT eGFP* mice (*n* = 27 cells from 5 mice) and *ChAT eGFP*;*Cdk5^f/f^* mice (*n* = 30 cells from 5 mice). Resting membrane potentials (RMP): *P*=0.2079, input resistance: *P*=0.1595, tau: *P*=0.2463, Cm: *P*=0.9008, unpaired two-tailed Student’s *t* test. Data represents mean ± SEM.


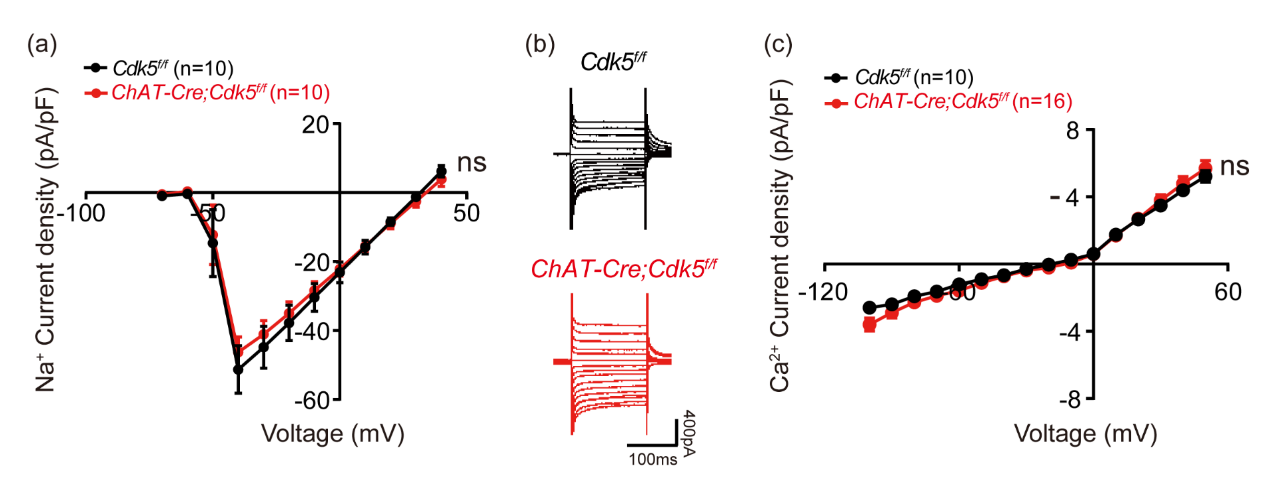


**FIGURE S4: Cholinergic neuron-specific *Cdk5* deficiency has no effect on Na^+^ currents and Ca^2+^ currents of striatal cholinergic neurons, related to FIGURE. 4.** (a) Summary of Na^+^ currents of striatal cholinergic neurons from *ChAT-Cre;Cdk5^f/f^;ChAT* *eGFP* mice (*n* = 10 cells from 4 mice) and *ChAT eGFP;Cdk5^f/f^* mice (*n* = 10 cells from 4 mice). *P*=0.76, two-way ANOVA followed by Tukey’s multiple comparisons test. Data represent mean ± SEM. (b) Representative calcium currents of striatal cholinergic neurons. C Calcium current density of striatal cholinergic neurons from *ChAT-Cre;Cdk5^f/f^;ChAT* *eGFP* mice (*n* = 16 cells from 3 mice) and *ChAT eGFP;Cdk5^f/f^* mice (*n* = 10 cells from 2 mice). *P*=0.6566, two-way ANOVA followed by Tukey’s multiple comparisons test. Data represent mean ± SEM.

**
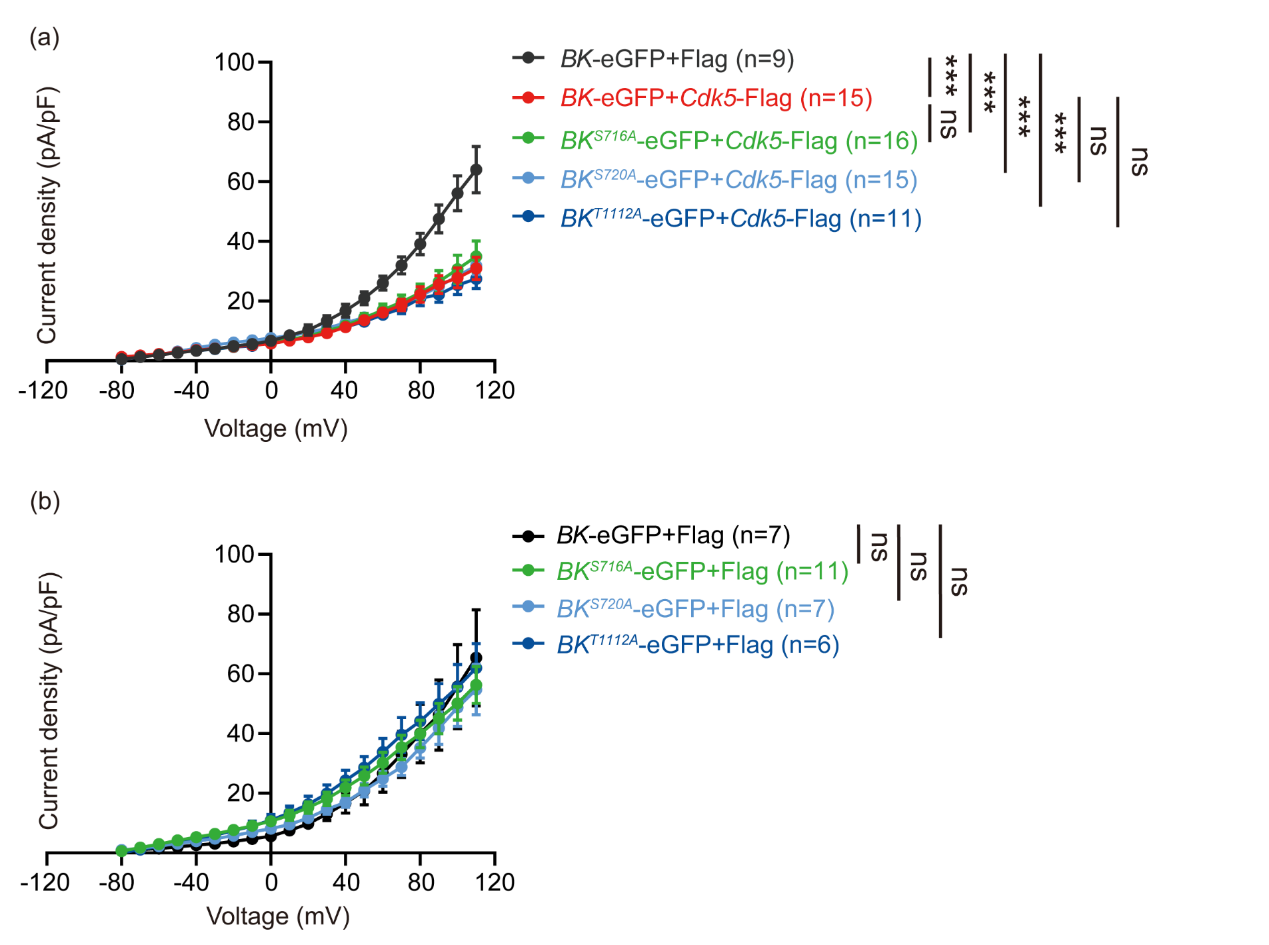
**

**FIGURE S5: Effects of BK mutations on BK-mediated currents, related to FIGURE. 5.** (a) Current-voltage relationships for BK currents in N2A cells co-transfected with BK mutants and *Cdk5*-Flag. BK-eGFP+Flag (*n* = 9), BK-eGFP+*Cdk5*-Flag (*n* = 15), BK^S716A^-eGFP+ *Cdk5*-Flag (*n* = 16), BK^S720A^-eGFP+*Cdk5*-Flag (*n* = 15) and BK^T1112A^-eGFP+ *Cdk5*-Flag (*n* = 11). (b) Current-voltage relationships for BK currents in N2A cells co-transfected with BK mutants and Flag. BK-eGFP+Flag (*n* = 7), BK^S716A^-eGFP+Flag (*n* = 11), BK^S720A^-eGFP+Flag (*n* = 7), BK^T1112A^-eGFP+Flag (*n* = 6). Analysis between group BK-eGFP+Flag and BK^S716A^-eGFP+Flag, *p*=0.4046; analysis between group BK-eGFP+Flag and BK^S720A^-eGFP+Flag, *P*=0.9543; analysis between group BK-eGFP+Flag and BK^T1112A^-eGFP+Flag: *p*=0.0621. All these above statistical analyses are two-way ANOVA followed by Tukey’s multiple comparisons test. Data represent mean ± SEM. ns, *P*>0.05, and *^***^P*<0.001.

**Supplementary Videos**

**Video S1.**

The *Cdk5^f/f^* mice exhibited no paw tremors.

**Video S2.**

Loss of *Cdk5* in striatal cholinergic neurons induced paw tremors in mice.
